# Immunogenicity of Recombinant Lipid-Based Nanoparticle Vaccines: Danger Signal vs. Helping Hand

**DOI:** 10.3390/pharmaceutics16010024

**Published:** 2023-12-23

**Authors:** Vladimir Temchura, Jannik T. Wagner, Dominik Damm

**Affiliations:** Institute of Clinical and Molecular Virology, University Hospital Erlangen, Friedrich-Alexander University Erlangen-Nürnberg, 91054 Erlangen, Germany; jannik.wagner@uk-erlangen.de

**Keywords:** lipid-based nanoparticles, liposomes, Toll-like receptor ligand, heterologous T cell help, intrastructural help, universal T cell epitope, immunogenicity, cellular immunity, vaccine antigen, adjuvants

## Abstract

Infectious diseases are a predominant problem in human health. While the incidence of many pathogenic infections is controlled by vaccines, some pathogens still pose a challenging task for vaccine researchers. In order to face these challenges, the field of vaccine development has changed tremendously over the last few years. For non-replicating recombinant antigens, novel vaccine delivery systems that attempt to increase the immunogenicity by mimicking structural properties of pathogens are already approved for clinical applications. Lipid-based nanoparticles (LbNPs) of different natures are vesicles made of lipid layers with aqueous cavities, which may carry antigens and other biomolecules either displayed on the surface or encapsulated in the cavity. However, the efficacy profile of recombinant LbNP vaccines is not as high as that of live-attenuated ones. This review gives a compendious picture of two approaches that affect the immunogenicity of recombinant LbNP vaccines: (i) the incorporation of immunostimulatory agents and (ii) the utilization of pre-existing or promiscuous cellular immunity, which might be beneficial for the development of tailored prophylactic and therapeutic LbNP vaccine candidates.

## 1. Introduction

While live-attenuated and recombinant antigen vaccines greatly contributed to the containment of multiple pathogens and even enabled the eradication of smallpox in the 20th century, the ever-increasing number of licensed nanoparticle-based vaccines is about to change the field of vaccinology in the 21st century [1,2]. Nowadays, vaccines have evolved into recombinant products with precise formulation and molecular definition. For non-replicating recombinant vaccines, a nanoparticulate structure that mimics viral properties greatly contributes to an increased immunogenicity in vaccinees [3].

**Figure 1:**
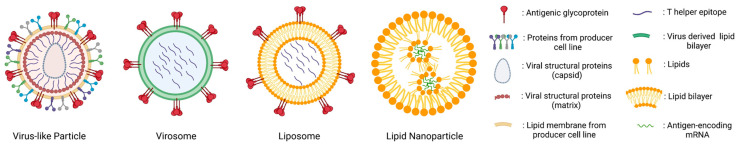
Overview of LbNPs. Displayed are different lipid-based nanoparticles and their components. Created with BioRender.com.

This review will focus on lipid-based nanoparticles (LbNPs) only and distinguishes between (i) enveloped virus-like particles (VLPs) that post-translationally self-assemble from viral subunits, (ii) virosomes, (iii) liposomes that contain a lipid bilayer with an aqueous cavity and (iv) lipid nanoparticles (LNPs) with a micelle-like monolayer structure in complex with internally loaded mRNA (Figure 1). For inorganic or polymer-based nanoparticles, we refer to excellent reviews from colleagues [4,5,6,7,8].

Self-assembling VLPs represent the vaccine delivery platform that is most similar to the biophysical properties of a prototype virus. Lentiviral enveloped VLPs were one of the most promising vaccine candidates against human deficiency virus (HIV-1) in the early 21st century since they resembled native virions presenting trimers of the surface glycoprotein (Env). However, they were never approved for clinical vaccine trials [9,10]. Due to various hurdles in the production and licensing of enveloped VLPs (reviewed in [11]), the focus of investigation recently shifted towards the development of tailored, synthetic nanoparticle-based platforms [12].

Virosomes combine the immunological benefits of “natural” enveloped VLPs with the advantages of controlled liposomal composition. Fundamentally, virosomes can be categorized as a subtype of LbNP subunit vaccines and exhibit lipid-anchored antigens on the surface of lipid vesicles. The lumen of virosomes, however, is free from the original viral structural proteins and nucleic acids. These synthetic nanoparticles are assembled in vitro by harnessing cell-free systems. Parts of their lipid membrane stem from purified viral membrane components (often derived from influenza A virus (IAV)) reassembling in the lipid nanoparticles with selected antigens of interest [13]. This feature ensures a tighter control of their composition in comparison to enveloped VLPs and provides the flexibility to adapt the particle to various types of antigens and adjuvants [14]. The licensed virosomal vaccines Inflexal^®^V and Epaxal^®^ against IAV and hepatitis A virus (HAV), respectively, demonstrated impressive immunogenicity and tolerability profiles [15].

Liposomes are spherical vesicles made of a lipid bilayer with an aqueous cavity. Antigens and other biomolecules can be conjugated to the liposomal surface or encapsulated in the inner cavity. In fact, liposomes are the first nanomedicine delivery platform that has effectively transitioned from theoretical design to clinical implementation [16]. Over the recent years, rapid advancements in modifying liposomes have been made by the incorporation of antigens, immunomodulators, adjuvants, and targeting molecules. This progress, coupled with the integration of innovative immunization devices, has transformed liposomes into a versatile and multifunctional delivery system (reviewed in [17]). To achieve effective immune modulation using liposome-based vaccines in vivo, the following key points must be taken into account: (i) the physicochemical characteristics of the liposomes, (ii) the choice of antigens and their integration (e.g., embedding, encapsulation, conjugation), and (iii) additional triggers for innate and/or adaptive immunity [18,19,20].

The liposomal formulation and the choice of lipid composition are easily adaptable for various approaches, i.e., the zeta potential of the nanoparticles may be switched from anionic to cationic by the exchange of only one lipid component [21]. In fact, several studies have shown that the surface charge of nanoparticles is decisive for efficient recognition and uptake by immune cells [22,23]. For example, a positive charge, which facilitates a beneficial interaction with the negatively charged cellular membrane, along with the interaction of APCs with the lipid head groups, seems to be a crucial element in triggering DC activation [18,24]. In contrast, anionic liposomes have been shown to possess tolerogenic properties [22,23]. They might mimic apoptotic bodies, which consequently inhibit DC maturation by contact [25]. Besides the influence on the zeta potential, the choice of lipid composition can already be a significant factor. Huang et al. observed that liposomes composed of bacterial lipids derived from *Deinococcus radiodurans* demonstrated enhanced vaccine efficacy compared to liposomes composed of widely used lipids like 1,2-Dioleoyl-sn-glycero-3-phosphocholine (DOPC) [26]. Additionally, the lipid composition may have an influence on the liposomal rigidity. APCs more readily envelop rigid particles than flexible ones, resulting in a more effective uptake [18,27,28].

Numerous research works and review papers highlighted and summarized the impact of particle size on the mechanisms of uptake by APCs, which could subsequently affect the processing and presentation of liposomal cargo to T cells [20,22,23]. It is feasible to introduce reactive groups into the liposomal surface layer via phospholipids, which enables the non-covalent or covalent conjugation of linear and conformational antigens and/or targeting antibodies on the liposomal surface [29,30,31,32,33,34]. Encapsulation of antigenic proteins or immunodominant peptides (epitopes) into vaccine liposomes might be essential. For the process of passive encapsulation, the volume captured within the liposome, which is believed to be a product of the liposome’s size and the overall lipid concentration, plays a significant role [35,36]. In contrast, electrostatically driven encapsulation might achieve a controlled and efficient vaccine production. The wide-ranging characteristics of peptides, such as their water solubility, hydropathicity, and isoelectric point, together with the composition of the liposome (especially the inclusion of charged membrane components), are key factors that influence the encapsulation process, which is primarily driven by electrostatic forces for hydrophilic peptides and proteins [21,35,37,38].

In comparison to liposomes, LNPs consist of an ionizable lipid monolayer. These particles form micelle-like structures that may incorporate antigen-encoding mRNA complexed with cationic phospholipids. The successful development and the recent licensing of LNP-based SARS-CoV-2 mRNA vaccines has greatly increased the interest of vaccine researchers in these vesicles [39,40].

Liposomes and LNPs have the least cytotoxic effects in comparison to other major types of nanoparticles (NPs) in various biomedical in vivo applications [40,41]. However, the efficacy profile of most recombinant LbNP vaccine formulations is not as high as that of live-attenuated vaccines and requires a series of injections to generate protective immunity. For some pathogenslike HIV-1,limited immunogenicity of the target immunogen and poor induction of neutralizing antibodies of the appropriate specificity are additional obstacles in vaccine development [42]. Therefore, the design of LbNP vaccines that are both safe and induce potent, long-lasting immune responses is a considerable challenge [43].

This review discusses two main approaches that affect the immunogenicity of LbNP vaccines: (i) the incorporation of immunostimulatory compounds for innate immunity and (ii) the harnessing of pre-existing or promiscuous adaptive cellular immunity. Utilizing these strategies might be beneficial for the development of tailored prophylactic and therapeutic LbNP vaccines.

## 2. Enhancement of LbNP Vaccine Efficacy via Toll-like Receptor Ligands

### 2.1. Toll-like Receptor (TLR) Ligands

The immune system possesses an inherent capability to identify molecular patterns carried by microorganisms. These patterns, known as pathogen-associated molecular patterns (PAMPs), activate specialized receptor sensors within cells and trigger a coordinated response to eliminate invading pathogens. In general, PAMPs are molecules that perform key functions for the microbes, but are not naturally found in the host organism. Examples are lipopolysaccharides (LPS) on the surface of Gram-negative bacteria, double-stranded RNAs (dsRNA) produced as part of viral replication, and flagellins that build bacterial flagella [44,45,46].

**Figure 2:**
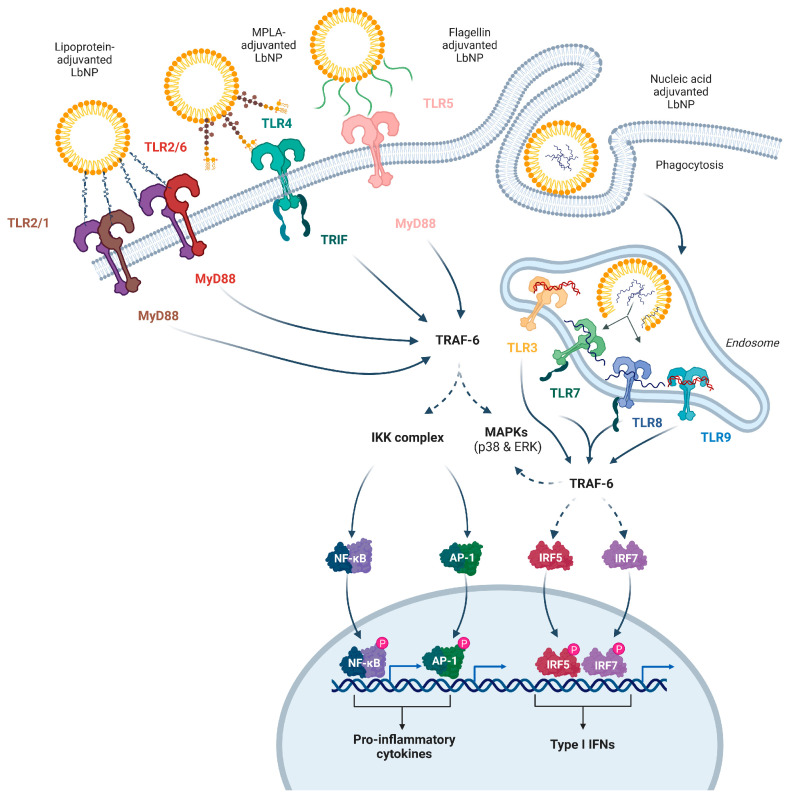
Toll-like receptors (TLRs) and their ligands. The extracellularly located TLR2 dimerizes with TLR1 or TLR6 and senses triacyl-lipoproteins or diacetyl-lipoproteins, respectively, and initiates TRAF-6 activation via MyD88 signaling. TLR4 and TLR5 are expressed on the cell surface. Upon binding of LPS (derivatives) or flagellin displayed on LbNPs, the receptors activate TRAF-6 induction via TRIF or MyD88, respectively. This triggers the initiation of the MAP-kinase (MAPK) and IKK complex cascade, which ultimately leads to the transcription of pro-inflammatory cytokines via the activation of NF-kB and AP-1. The phagocytic uptake of LbNPs containing nucleic acid-based adjuvants triggers the activation of TLR3, TLR7/8, or TLR9 after endosomal release. Here, TLR3 binds dsRNA, TLR7/8 ssRNA, and TLR9 CpG-rich DNA. This mediates the downstream activation of TRAF-6, which, on the one hand, triggers the induction of pro-inflammatory cytokines, as described for TLR4 and TLR5. On the other hand, endosomal TRAF-6 induction leads to an additional upregulation of Type I IFNs via the IRF5/IRF7 pathway. Created with BioRender.com.

The TLR family is a well-characterized group of innate signaling receptors that respond to a variety of PAMPs. TLR1, TLR2 and TLR6 form heterodimers and recognize lipopeptides. TLR4 senses LPS and derivatives like monophosphoryl lipid A (MPLA), and TLR5 binds to flagellins. The endosomal TLR3 detects dsRNA, while TLR7 and TLR8 recognize single-stranded RNA (ssRNA), and TLR9 responds to CpG-rich nucleotide patterns [45,47,48,49,50,51]. The different TLRs are either located on the cell membrane or inside endosomes depending on the source of the PAMPs, i.e. LPS shed from bacteria is picked up by lipopolysaccharide-binding protein (LBP) and transported to TLR4 on the cell membrane, whereas the viral RNA sensors (TLR3, TLR7, TLR8) are located in endosomes of the host cells (Figure 2) [52,53]. Upon ligand binding, the downstream signaling pathways activate the transcription and secretion of type I and II interferons as well as immunostimulatory cytokines and chemokines. TLR-based adjuvants can trigger a T helper type 1 (Th1), Th2, or Th17 biased immune response. For example, a Th1-prone response gets activated by binding of an adjuvant to TLR3, TLR4, TLR7, TLR8, or TLR9 [54,55,56].

Poor TLR stimulation might lead to inappropriate immune responses after vaccination. An experimental inactivated respiratory syncytial virus (RSV) vaccine lacking proper TLR signaling did not protect infants from infection, but instead exacerbated the symptoms of natural RSV exposure in the vaccinees [57,58]. However, Delgado et al. suggested that inactivated RSV vaccines may be rendered safe and effective by the incorporation of TLR agonists in their formulation [59].

In this chapter, we will discuss several TLRs and their respective agonists that are currently involved in experimental or licensed LbNP vaccines in detail (Figure 2).

### 2.2. TLR2 Heterodimers and Their Antagonists

The discovery and cloning of TLR2 were initially reported in 1998 [60]. The expression of TLR2 has been observed in immune cells, as well as endothelial and epithelial cells [61]. It is the sole TLR known to date that can form operational heterodimers with more than two other TLR types (Figure 2). The idea of TLR2 operating as a functional homodimer has been suggested by Jin et al., but there is currently no proof that it initiates a signaling sequence in humans and/or mice [62]. TLR2 signaling is initiated by ligand-induced dimerization and was described in detail elsewhere [63]. Briefly, ligand-induced dimerization of the essential cytoplasmic TIR domains triggers a cascade of phosphorylation events that vary depending on whether it was TLR2/1 or TLR2/6 stimulated. These signaling cascades result in the MyD88-dependent activation of pro-inflammatory transcription factors such as NF-κB and AP-1 (Figure 2).

The ability of TLR2 to form heterodimers not only diversifies downstream signaling cascades but also facilitates the recognition of a wide variety of PAMPs from structurally different bacterial compounds [64]. Many TLR2 agonists identified so far (e.g., glycolipids, lipoproteins, lipopolysaccharides) contain a hydrophobic component, making them an attractive adjuvant for liposomal vaccine formulations. One of the most promising lipid moieties for use in such vaccines is S-[2,3-bis(palmitoyloxypropyl)]cysteine (Pam2Cys), a simple synthetic metabolizable lipoamino acid derived from the lipid component present in Mycoplasma [65]. Although Pam2Cys only consists of a cysteine, a thioglycerol and two fatty acid residues, it signals via the TLR 2/6 pathway, activates dendritic cells, and enhances both humoral and cellular adaptive immune responses [66].

The swift advancement of mRNA vaccines in recent years has been remarkable. These vaccines, particularly those aimed at SARS-CoV-2, have rapidly moved from the laboratory to clinical application, playing a significant role in combating the COVID-19 pandemic. Concurrently, extensive research and development efforts are underway for mRNA vaccines targeting various cancers and other infectious diseases. Recently, Gu et al. investigated the enhancement of the efficacies of mRNA vaccines with Pam2Cys. The adjuvant was incorporated into mRNA-LNPs to achieve co-delivery with mRNA. Adjuvanted mRNA-LNPs effectively modulated the immune environment in the draining lymph nodes, leading to the production of IL-12 and IL-17, among other cytokines. The presentation of antigens by DCs resulted in significantly enhanced anti-tumor responses in both prophylactic and therapeutic tumor models, dependent on CD4+ and CD8+ T cells. This process also led to the establishment of memory anti-tumor immunity. Furthermore, the vaccine stimulated a considerably stronger humoral and cellular immunity in a surrogate COVID-19 prophylactic model. Importantly, these new Pam2Cys-adjuvanted mRNA vaccines demonstrated promising initial safety profiles in murine models [67].

Since the onset of the SARS-CoV-2 pandemic, mucosal vaccination routes also have become a point of interest within the scientific community. However, the lack of reliable mucosal adjuvants is still one of the major challenges in the development of mucosal recombinant vaccines. Naciutae et al. developed a liposomal vaccine formulation for oral delivery consisting of a long tumor peptide and Pam2Cys in order to stimulate local and systemic immune responses. Lipid-based vaccines (i) induced expansion of lymphocyte populations in mesenteric lymph nodes of naïve (tumor-free) mice and (ii) reduced the growth of tumor cells in the gastrointestinal tract of mice with colorectal cancer [68].

To trigger the TLR2/TLR1 pathway, triacylated lipopeptides such as Pam3CysSerLys4 (Pam3CSK4) and their derivatives have been tested in experimental vaccines [69]. Bal et al. clearly demonstrated that co-encapsulation of Pam3CSK4 together with an antigen in cationic liposomes modulates the type of immune response. First of all, liposome-based Pam3CSK4 formulation induced superior DC maturation compared to the free adjuvant. Surprisingly, co-encapsulation of ovalbumin and Pam3CSK4 in cationic liposomes did not influence the total anti-ovalbumin IgG titers compared to the antigen/adjuvant solution but shifted the IgG1/IgG2a balance [70].

In the context of versatile liposomal constructs for co-delivery of tumor peptide epitopes in combination with TLR ligands, Thomann et al. performed a direct comparison between synthetic TLR2/1 and TLR2/6 agonists. It was demonstrated that the TLR2/6 agonists (Pam2CAG and Pam2CGD) were more efficient than the TLR2/1 agonist (Pam3CAG) for the therapeutic anti-tumor vaccination [71].

Besides synthetic lipopeptides, various proteins, including lipoproteins and glycoproteins, can trigger TLR2 [64]. An elegant example of liposomal vaccine development was demonstrated by Banerjee et al. Porin of *Shigella dysenteriae* was incorporated into liposomes (PIL). On the one hand, liposomes served as an attractive vehicle to embed the porin in the liposomal bilayer and present it to the adaptive immune system. On the other hand, the porin molecule in its native form could serve as a TLR2 adjuvant. Indeed, PIL triggered the TLR2/TLR6 pathway on DCs, leading to their maturation. PIL-stimulated DCs provided activation and Th1 polarization of allogeneic CD4+ T cells, thereby successfully bridging innate and adaptive immunity [72].

Thus, lipopeptides are the most broadly used ligands for TLR2 heterodimers in vaccine development. The process of integrating variable synthetic TLR2 ligands into liposomal structures is quite straightforward, which simplifies the manufacturing of entirely synthetic LbNP vaccines. TLR2 adjuvants stimulate DCs, thereby enhancing cell-mediated reactions and modulating antibody levels, which could be advantageous in warding off infections or eradicating tumors. However, the capability of vaccines with TLR2 adjuvants to generate CTL responses is not particularly notable. Therefore, the merits of focusing on TLR2 for vaccine development remain a contentious issue, calling for additional research (rev in [73]).

### 2.3. TLR3-Activating Adjuvants

TLR3 is mainly expressed in endosomal compartments of myeloid DCs (mDCs) and recognizes viral dsRNA [74]. Synthetic dsRNA analogs like NexaVant or polyriboinosinic:polyribocytidylic acid (poly(I:C)) also bind to TLR3, which induces TRIF/TRAM pathway-dependent signaling, resulting in the secretion of inflammatory cytokines and IFN-β [75,76]. Furthermore, poly(I:C) is able to activate intracellular receptors like retinoic acid-inducible gene I (RIG I) or melanoma differentiation-associated gene 5 (MDA-5), resulting in adjuvant activity [77]. In general, TLR3 ligands induce a potent activation of dendritic cells, which promotes the elicitation of strong Th1 CD4+ T cell, CD8+ T cell and NK cell responses. Major flaws that should always be considered with regard to poly(I:C)-adjuvanted formulation are its low stability and toxic side effects. Once applied, it may be degraded by serum nucleases. This can not be counteracted by increasing the dosage of poly(I:C) since it is not well tolerated in high doses [78].

The encapsulation of poly(I:C) inside liposomes protects them from degradation in vivo and has shown promising results in several animal studies [79,80,81,82]. The intradermal injection of liposomes that incorporated poly(I:C) together with a synthetic peptide harboring a model CTL epitope resulted in stronger induction of peptide-specific CD8+ T cell responses compared to the control groups, which included poly(I:C)-adjuvanted soluble peptide or peptide-loaded liposomes mixed with poly(I:C). The poly(I:C)-encapsulating liposomal formulation resulted in a 25-fold increased peptide-specific CD8+ T cell frequency in comparison to the soluble peptide control. Although the priming of CD8+ T cell responses was improved in mice immunized with adjuvanted liposomes irrespective of the way poly(I:C) was delivered (encapsulated or mixed), the authors observed a significant difference in the functionality of the induced T cells between the peptide/poly(I:C)-loaded liposomes and the peptide-loaded liposomes + poly(I:C) formulations [83].

In order to analyze humoral immune responses, poly(I:C) was co-encapsulated either with a model antigen (ovalbumin (OVA) [79]) or with a pathogen-derived antigen (Diphtheria Toxin (DT) [81]). OVA/poly(I:C)-loaded liposomes significantly increased the antigen-specific IgG2a response compared to soluble OVA and poly(I:C) solutions in immunized mice [79]. Surprisingly, both liposomal DT/poly(I:C) formulations and liposomes that encapsulated either poly(I:C) or DT induced higher IgG2a titers compared to the control groups. Furthermore, DT and poly(I:C) individually encapsulated into liposomes resulted in similar IgG2a titers than DT/poly(I:C) co-encapsulated into liposomes [81].

In a study by Hu and colleagues, the liposome-encapsulated administration of poly(I:C) was able to enhance the immune response against Dengue virus. Here, the vaccinated mice showed high antibody levels and, upon virus challenge, decreased loss of body weight and fewer viral copies in the brain tissue [82].

Thus, different liposomal antigen/poly(I:C) formulations might, in general, be suitable for (i) therapeutic vaccine candidates against cancer since antigen/poly(I:C) co-encapsulation appeared to be effective regarding the induction of cytokine-secreting CD8+ T cells with increased killing capacity [83,84], and for (ii) prophylactic antiviral vaccine candidates, because a strong Th1-biased immune response during viral infections or after vaccination was demonstrated to be beneficial for the induction of antiviral antibody responses [85,86].

### 2.4. TLR4-Activating LPS Derivatives

TLR4 is expressed by the majority of circulating immune cells but was first described in macrophages and mDCs [87,88]. TLR4 signals in MyD88-dependent and TRIF-dependent ways, inducing a robust IL-12-mediated secretion of type I interferons (IFNs) and a strong Th1-biased T cell and humoral immune response [89]. Within DCs, the activation of TLR4 leads to a reorganization of lysosomal distribution dependent on Rab34. This rearrangement results in a delay in antigen degradation, temporarily boosting cross-presentation. Consequently, this optimization contributes to the effective priming of CD8+ T cell responses against pathogens [90].

A variety of TLR4 ligands that are derived from fungi, viruses, parasites, endogenous ligands and bacterial products have been characterized [91,92,93,94,95]. The major agonists that were described first and remain the best characterized are lipopolysaccharides from Gram-negative bacteria [91,96].

MPLA, manufactured from *Salmonella enterica* endotoxin, has been widely used for clinical applications and replaced alum as the predominantly accepted adjuvant for licensed vaccines and therapeutics [97]. Purification of MPLA can easily be achieved by acid hydrolysis of LPS followed by centrifugation and washing of the precipitated heptose-free MPLA [98]. Removal of the glucosamine-1-phosphate group from the polar head of lipid A results in the generation of MPLA. The absence of glucosamine-1-phosphate reduces the toxic effects in vivo compared to LPS, but MPLA is still recognized by TLR4 [99,100]. Since MPLA contains a negative charge, MPLA liposomes usually bear an anionic zeta potential. MPLA liposomes can easily be generated at room temperature and are ready to use as adjuvants [101].

MPLA/TLR4 binding induces a strong Th1-biased immunity as well as the secretion of pro-inflammatory cytokines and promotes antigen-specific CD8+ T cell responses, which has been reported for liposomal vaccine formulations against parasitic (*Leishmania*) [102], bacterial [103,104] (*Mycobacterium tuberculosis*) and viral (HIV-1, Ebola virus (EBOV), SARS-CoV-2) pathogens [105,106,107,108].

Although these studies were performed with lipid vesicles carrying antigens in the aqueous core and MPLA embedded in the vesicle walls, co-presentation of B cell antigens and MPLA on the LbNP surface also improves humoral immune responses. For example, Ingale et al. manufactured MPLA liposomes that displayed the HIV-1 surface glycoprotein (Env) on the surface [30]. The authors demonstrated higher anti-Env antibody levels, a stronger induction of neutralizing antibody responses, and a more dominant germinal center formation in rabbits immunized with Env/MPLA-liposomes compared to control animals that received soluble Env trimers only. Hanson et al. managed to elicit antibody responses against the membrane-proximal external region (MPER) of the HIV surface glycoprotein Env when the protein subunit was anchored on the surface of liposomes. Immunization with soluble MPER + oil-in-water adjuvants or alum did not elicit MPER-specific antibodies. High-titer anti-MPER IgG responses could be induced when MPLA was additionally incorporated in the MPER-liposomes [109].

MPLA was also used as an adjuvant in virosomal vaccines. Kamphuis et al. utilized RSV virosomes that incorporated MPLA adjuvant to boost immunogenicity and direct the immune response towards a Th1 phenotype. The inclusion of MPLA amplified the overall immunogenicity of the virosomes in comparison to virosomes without adjuvants in mice. The intramuscular delivery of the vaccine resulted in the production of RSV-specific IgG2a levels comparable to those produced by the animals exposed to live RSV [110]. Dong et al. created a modified virosomal platform that comprised the membrane lipids and proteins of the influenza virus. MPLA was additionally integrated into the membrane and the conserved IVA nucleoprotein was conjugated to the membrane. In vitro, such virosomes containing MPLA triggered a more robust activation of APCs than virosomes without adjuvants. In vivo, virosomes adjuvanted with MPLA effectively generated antigen-specific antibody responses and effectively primed CTLs [111].

Vaccines containing MPLA-LbNPs as a part of the licensed Adjuvant System 01 (AS01) have been licensed in some countries and included in vaccination programs. AS01, a liposome-based adjuvant, is utilized in two approved vaccines: RTS,S/AS01 (against malaria) and Shingrix (a recombinant zoster vaccine). This adjuvant comprises two immunostimulatory agents: MPLA and QS-21 (a saponin molecule derived from the bark of *Quillaja saponaria Molina*). MPLA and QS-21 demonstrate a synergistic effect, working together to amplify antigen-specific responses. This synergy operates via a mechanism that involves the early induction of IFN-γ in the draining lymph node, subsequently fostering a robust Th1 response [112]. In general, MPLA is the most favorable TLR agonist for the adjuvantation of HIV-1 Env immunogens since it was shown to have no negative influence on the stabilized pre-fusion conformation of this trimeric antigen, unlike other adjuvants that slightly interfered with the immunogen structure (CpG, imiquimod) or even resulted in the dissociation of the trimeric complex (alum) [113]. Notably, MPLA-liposomes are the adjuvant of choice for two phase-1 prophylactic HIV vaccine studies using Env trimers as main immunogens (NCT03816137, NCT03699241).

Another LPS derivative with agonistic TLR4 adjuvant activity is deacylated lipooligosaccharide (dLOS). dLOS consists of a core oligosaccharide lacking the terminal glucose residue, a glucosamine disaccharide with two phosphate groups, and two N-linked acyl groups. dLOS-induced cytokine production in mouse peritoneal macrophages is comparable to MPLA but exhibits superior activation capabilities in human monocytes and DCs [114]. In preclinical animal studies, the adjuvant CIA09, comprising cationic liposomes and dLOS, effectively boosted antibody and cell-mediated immune responses against recombinant tuberculosis antigens, inactivated Japanese encephalitis vaccine (JEV), and recombinant varicella-zoster virus (VZV) glycoprotein E (gE) antigen [115,116,117,118].

In summary, LbNPs containing LPS derivatives have shown considerable promise as adjuvants for immunization. In comparison to LPS, they have a decreased toxicity and, therefore, are safe for use in humans.

### 2.5. Flagellin—A TLR5 Agonist

TLR5 is found on numerous cell types, such as lymphocytes, macrophages, monocytes, neutrophils, NK cells, DCs, epithelial cells and stromal cells in the lymph nodes [119]. It identifies bacterial flagellin, which is a structural element of the flagellum—a movement apparatus primarily linked with Gram-negative bacteria—as an extracellular PAMP and activates both the MyD88-dependent signaling pathway and NF-κB-mediated production of pro-inflammatory cytokines [120]. Flagellin is a potent activator of the innate and adaptive immune system and has shown its tremendous potency as an adjuvant, either in the context of a fusion protein or by co-administration with antigens (reviewed in [119] and in [121]). Truncated, less inflammatory forms of flagellin can be used to avoid side effects.

More than a decade ago, it was demonstrated that the adjuvant properties of flagellin could be conveniently harnessed to target liposome-associated antigens to APCs, as well as to induce potent antigen-specific and anti-tumor immunity. Antigen-functionalized liposomes coated with flagellin-related peptides could effectively enhance the immune response against tumor cells, leading to a reduction in tumor growth [122].

Given that TLR5 is widely expressed in lung and intestinal epithelial cells, flagellin has garnered significant interest as a mucosal adjuvant [123]. Chimeric VLPs of IAV (H5N1) that bear membrane-anchored flagellin (FliC-VLP) were applied to mice both intramuscularly and orally. These mice exhibited stronger humoral and cellular immune responses compared to those receiving H5N1 VLPs without flagellin. Mice that received an oral immunization with Flic-VLPs demonstrated significant protection against lethal exposure to both homologous and heterologous H5N1 influenza viruses, while mice that received oral immunization with VLPs lacking flagellin invariably fell victim to the infection. Additionally, mice orally immunized with FliC-VLPs displayed virus-specific IgG titers tenfold higher than their counterparts [124]. In a guinea pig model, HIV-1 VLPs with membrane-anchored flagellin induced enhanced antibody responses by either systemic or mucosal vaccination, as demonstrated by high levels of HIV-specific serum IgG as well as mucosal IgG and IgA. VLPs incorporating full-length flagellin were more effective in inducing systemic responses, while VLPs containing truncated forms of flagellin were more effective in inducing mucosal IgA responses [125].

Unlike the majority of TLR ligands, flagellin, being a protein, has the ability to elicit an immune response directed against itself. Barnowski et al. observed in B cell-targeting lentiviral VLP vaccines that flagellin acts as an antigen, potentially overwhelming the antibody response to a less immunogenic antigen. However, when paired with a potent immunogen, the adjuvant activity of flagellin might overshadow its own immunogenicity [126].

In summary, the use of flagellin as a TLR5-activating adjuvant in LbNP vaccines has shown promising results in enhancing the immune response and inducing anti-tumor immunity. Further research in this area could lead to the development of more effective vaccines and immunotherapeutic strategies. Because of its potential immunogenicity, concerns about diminished efficacy and possible reactogenicity after repeated administration exist. Immunization with truncated flagellin derivatives while preserving the TLR5-mediated immunomodulatory activity should be the most reasonable option for clinical applications [127,128].

### 2.6. Adjuvants Interacting with TLR7 and TLR8

TLR7 and TLR8 (CD288) are both expressed in NK cells, neutrophils, monocytes, macrophages, eosinophils and Langerhans cells. B cells and plasmacytoid DCs (pDCs) contain TLR7 only, while T cells only express TLR8 [129]. TLR7 and TLR8 are located intracellularly within endosomal compartments. Activation of TLR7 and TLR8 initiates signaling pathways involving MyD88/Mal, NF-κB and IRF7, leading to the secretion of pro-inflammatory cytokines, chemokines, and other mediators. In DCs, activation of TLR7/TLR8 promotes cell maturation, expression of co-stimulatory molecules (CD40, CD80, and CD86), enhanced antigen presentation, and secretion of Th1 pro-inflammatory cytokines (IL-12, TNF-α and IFN-α). TLR7 signaling results in the secretion of immunoglobulins, IL-6, and TNF-α by B cells, as well as IFN-γ by NK cells [130,131]. Activation of TLR8 enhances T cell proliferation, IFN-γ, IL-2 and IL-10 production, memory T cell activation, and reduces CD4+ Treg-mediated immunosuppression [132].

The ligands for TLR7 and TLR8 include ssRNA enriched for poly-U or poly-GU sequences, synthetic imidazoquinolinamines like imiquimod (R-837) and resiquimod (R-848), as well as guanosine analogs such as loxoribine [133,134]. While resiquimod and its analogs have proven to be effective as vaccine adjuvants in various murine models, it is evident that these specific molecules may not be universally optimal adjuvants, especially when the TLR agonist is mixed with an antigen and administered via conventional injections [135]. Most notably, R-848 showed a poor tolerability profile in humans as well as common systemic side effects. These side effects included injection site reactogenicity and flu-like symptoms (fever, headache, and malaise) that correlated with systemic immune activation, i.e., as shown by high concentrations of numerous cytokines in the blood [136]. Additionally, in contrast to adjuvants with a “depot effect”, the small, synthetic TLR7/TLR8 agonist molecules are rapidly distributed throughout the body after subcutaneous injection and may cause systemic rather than localized stimulation [137,138]. Therefore, nanoparticulate formulations (e.g., liposomal encapsulation of TLR7/8 agonists) might improve immunogenicity due to restricted adjuvant distribution and prolongation of activity in draining lymph nodes [138,139].

By addition of an 18-carbon chain to 3M imidazoquinoline (IRM), Smirnov et al. created a molecule called 3M-052, which is more amenable to incorporation in lipid-based formulations such as liposomes or emulsions [140]. Later, Van Hoeven et al. developed a formulation for 3M-052 liposomes as an adjuvant for pandemic influenza vaccines. The modified 3M-052 liposomes increased the protective capacity of H5N1 antigens, promoting the broadening of antibody responses, antigen dose sparing, and protection in pre-clinical studies [141].

So far, TLR7/TLR8 agonists have not been clinically approved as vaccine adjuvant components [142]. Nevertheless, various formulations of vaccines adjuvanted with TLR7/TLR8 agonists are currently undergoing different stages of clinical trials. The adaptive response elicited in these adjuvant studies is marked by a Th1-like phenotype, characterized by the production of IFN-γ by CD4+ cells and IgG2 by B cells, along with a simultaneous suppression of Th2 immunity. Continued assessment and refinement of these TLR7/TLR8 adjuvant strategies, including potential dose-sparing effects, reduced reactogenicity profiles, and thorough examination of long-term safety and efficacy outcomes, are undeniably warranted [136].

### 2.7. TLR9-Dependent Adjuvants

In humans, TLR9 (CD289) is found in intracellular endosomal compartments of immune cells, especially in B cells and pDCs [143]. TLR9 signaling via the MyD88 pathway, involving IRAK and TRAF-6 but not Mal, triggers the production of Th1-type pro-inflammatory cytokines (IL-1, IL-6, IL-12, IL-18, TNF-α, and IFN-γ), upregulation of CD40, CD80, CD86, and MHC molecules on the APC surface, enhanced antigen processing and presentation, as well as CD8+ T cell responses [144,145]. Notably, IL-12 and type I IFNs induced in pDCs via TLR9 contribute to robust Th1-type immunity and CD8+ CTL cytotoxicity, while B cell activation dependent on TLR9 leads to increased antigen-specific humoral responses and IgG class switching [146,147].

The ligands for TLR9 include bacterial and viral DNAs containing unmethylated CpG motifs, as well as synthetic oligodeoxynucleotides (ODN) expressing CpG motifs [148]. Synthetic TLR9 ligands, which maintain the immunostimulatory activity of bacterial DNA, are categorized into three main classes based on their structure, biological properties, and their ability to activate immune cells in vitro [149,150].

TLR9 agonists have been extensively studied in pre-clinical and clinical studies [151,152,153,154]. ODN adjuvants were tested in vaccine models targeting malaria, hepatitis B virus (HBV), IAV, anthrax, and SARS-CoV-2 [155,156,157,158,159]. CpG-ODN triggered a potent antibody response to the malarial Apical Membrane Antigen 1 (AMA1) and the Merozoite Surface Protein 142 (MSP142) [160]. In the context of HBV, a B-type CpG-ODN known as CPG 7909 amplified specific, long-term antibody responses to the Engerix-B^®^ vaccine (a recombinant HBsAg vaccine absorbed on alum) compared to Engerix-B^®^ alone [161]. Another CpG-ODN, the 1018 immunostimulatory sequence (ISS), has been demonstrated to enhance the efficacy of the HBV vaccine Heplisav^®^ with minor local side effects [162]. Conversely, the inclusion of CpG 7909 in the influenza vaccines Fluarix^®^ is considered less impactful, although it enhanced IFN-γ secretion and was well-tolerated, providing an advantage for reducing the vaccine dosage [157].

Since the utilization of nanoparticle-based vaccines has increased over the last decade, multiple studies have reported liposomal vaccine candidates that co-encapsulated both CpG-ODN and the immunogen of choice (OVA as a model system; Listeriolysin O of *Listeria monocytogenes*; a recombinant *Leishmania* Stress-Inducible Protein 1). All immunizations resulted in a strong Th1 response, mediated through a combination of liposomal delivery of antigen and CpG-ODN to the cytoplasm and endosomal uptake of the whole liposomes, triggering the TLR9 pathway. This also resulted in a robust CTL response, the generation of IFN-γ-secreting CD4+ and CD8+ T cells, and the production of Th1-type antibodies. In comparison to control groups, the total antibody levels were not significantly different, but a shift in the IgG1/IgG2a ratio to an IgG2a predominance was observed [70,163,164,165,166,167].

Based on the strong immunogenicity of liposomal CpG-ODN/antigen formulations, they can be considered an effective vaccine platform for the induction of antiviral immunity.

To sum up this chapter, TLR ligands are small biomolecules of different properties and origins that can easily be incorporated into LbNP vaccines and strongly promote humoral immune responses. The TLR4 agonist MPLA, in particular, is already the adjuvant of choice in a multitude of clinically licensed vaccines or part of complex adjuvant systems. Synthetic ssRNA-based TLR7/TLR8 agonists are currently in the process of being licensed for clinical vaccines. In general, TLR agonists already replaced alum as the predominant adjuvant in licensed vaccines. LbNPs with a lipid bilayer and aqueous cavity inherit the ideal properties to act as carriers for TLR delivery in combination with the immunogen of choice. Lipid-based TLR agonists such as MPLA can be incorporated in the bilayer of LbNPs, while soluble small TLR molecules like CpG or ssRNA may be encapsulated inside the nanoparticle. Such combinations of polyfunctional carriers and state-of-the-art adjuvants will shape vaccinology in the 21st century.

## 3. Improvement of Humoral Immune Responses via Heterologous Cellular Immunity

Since classical adjuvants and TLR agonists primarily target innate immunity and may cause undesirable side effects, a bolder way to enhance the acceptance of novel vaccines would be to induce robust and potent humoral immune responses without the need for any adjuvants in the formulation. Another challenge faced by recombinant protein vaccines is the potential lack of immunodominant epitopes, which can lead to suboptimal T cell help despite appropriate adjuvantation and, consequently, low-magnitude and low-affinity antibody responses. For LbNP formulations utilizing recombinant proteins, we present another possibility of adjuvant-free vaccine design that greatly influences and modifies humoral immune responses–the utilization of pre-existing or promiscuous cellular immunity to provide help for B cells specific for the antigen of interest. In the following chapter, we introduce two major routes to achieve this.

### 3.1. Intrastructural Help Harnessing Pre-Existing Immunity

A number of licensed live-attenuated vaccines, such as the measles virus vaccine, induce strong antibody responses with a plasma half-life of virus-specific IgG levels in excess of 100 years [168]. In contrast, antibody levels induced by Tetanus vaccines or experimental HIV vaccines decline relatively rapidly with a half-life of 11 years and less than one year, respectively, even if the vaccine antigen is delivered in the presence of adjuvants (reviewed in [169]). The prevailing hypothesis for these striking differences is that the strength of the signals an antigen-specific B cell receives during the germinal center (GC) reaction determines the half-life of the antibody response. In particular, signals from B cell receptor crosslinking, stimulation by T follicular helper (Tfh) cells, and/or triggering of pattern recognition receptors (PRRs) determine the numbers and the activity of long-lived plasma cells that ultimately derive from one B cell. In addition, these signals also determine the IgG subtype response and, thus, define the Fc-effector functions of the antibodies [170,171,172,173]. Consequently, the quality of the antibody response and its duration is already imprinted during the induction and affinity maturation phase.

**Figure 3:**
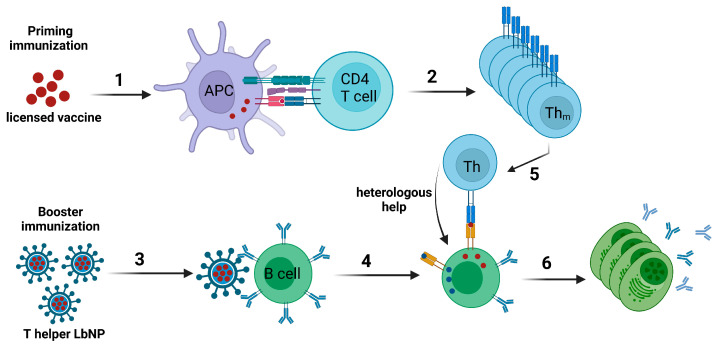
Intrastructural help. (1) Upon priming with a licensed vaccine, naïve CD4+ T cells bind corresponding peptides complexed on MHC-II molecules of APCs. (2) This interaction leads to the proliferation and expansion of the CD4+ T cells with the following differentiation into CD4+ T helper memory (Th_m_) cells. (3) T helper LbNPs, carrying viral glycoproteins on the surface and peptides from licensed vaccines inside, administered as booster immunization, are taken up in a BCR-dependent manner by glycoprotein-specific B cells. (4) After LbNP processing, peptides derived from heterologous proteins are presented on MHC-II molecules of the B cells. (5) These peptide/MHC-II complexes are recognized by previously induced heterologous T helper (Th) cells. (6) Subsequently, these non-cognate T cells provide help for the B cells, which leads to differentiation in plasma cells and the effective secretion of glycoprotein-specific antibodies. Created with BioRender.com.

The concept of intrastructural help (ISH), which was first described for influenza virus [174] and more recently for HIV VLPs [175], can be used to achieve a modulation of humoral immune responses by harnessing pre-existing cellular immunity specific for heterologous epitopes. Accordingly, a naïve B cell specific for the surface protein of a virus would take up the entire virion or VLP via a B cell receptor-dependent mechanism and present peptides derived from both the surface protein as well as from other proteins encapsidated in the particle on MHC-II molecules. Thus, T helper cells specific for the non-surface proteins of the particle may provide help for the B cell to elicit antibody responses against the surface protein (reviewed in [176]). Extending this concept with regard to the development of a widely applicable vaccine platform, one would generate “T helper LbNPs” that display the vaccine immunogen of choice on their surface and contain T helper cell epitopes from efficacious licensed vaccines inside [29,177]. B cells specific for the vaccine antigen on the surface of the LbNPs should internalize the entire nanoparticles and subsequently present the encapsulated peptide epitopes to T helper cells specific for and previously induced by the licensed vaccines (Figure 3).

Liposomes seem particularly well-suited as a vaccine platform in the context of ISH. Hydrophilic T helper cell epitopes could readily be encapsulated inside the liposomes by passive inclusion during liposomal formation or by electrostatically driven approaches that optimize the buffer condition as well as the lipid and peptide concentrations to actively promote encapsulation [21]. Alternatively, whole immunodominant protein antigens may also be encapsulated [178]. Furthermore, the lipid membrane provides a barrier for recognition of the T helper cell epitopes (peptides or proteins) present in the aqueous cavity by antibodies and B cell receptors, which prevents the T helper cell epitopes from distraction by the specific humoral immune response [179].

In addition, the vaccine antigen can be coupled to the surface of LbNPs in an ordered array that displays repetitive epitope structures for improved B cell recognition and activation via crosslinking of the BCRs [30]. Especially for HIV-1 Env, a multitude of conjugation strategies that maintain the pre-fusion conformation of the protein were established [31,180,181,182]. Since there is (i) an urgent need for a prophylactic HIV vaccine and (ii) Env—as a weak immunogen—only induces a particularly short-lived antibody response, when administered as an adjuvanted soluble antigen, HIV-1 is a perfect candidate to monitor enhancement of Env-specific antibody levels by ISH [169]. In order to induce ISH effects, the heterologous T helper epitope does not need to be encapsulated inside the nanoparticles. It may also be displayed on the surface alongside the antigen of interest. However, this may lead to a weakened phenotype of the immune response modulation against the antigen of interest if the B cell response to the heterologous epitope is too dominant [126].

Hills et al. were among the first to exploit pre-existing immunity in order to improve the immune response against an antigen of interest via liposomal vaccines in the context of ISH. As a *proof-of-concept*, the authors encapsulated OVA-derived OT-II peptide in liposomes that were surface-functionalized with the malarial circumsporozoite (CSP) antigen from *Plasmodium falciparum*. Mice were primed with OT-II peptide for the generation of OT-II-specific CD4+ T cell responses. The animals were then boosted with CSP/OT-II-liposomes (ISH group). Control groups received either a mock prime or empty CSP-liposomes as booster immunizations. The ISH mice showed a faster and higher anti-CSP humoral immune response with stronger avidity of the antibodies compared to the control groups. In particular, anti-CSP IgG2c antibodies, which are known to have highly antiviral Fc effector functions, were rapidly elicited and reached higher levels. In a translational follow-up experiment, mice were infected with murine cytomegalovirus (MCMV) and boosted with CSP-liposomes that encapsulated MCMV m09_133–147_ peptide. Likewise, anti-CSP IgG1, IgG2b, and IgG2c were significantly upregulated compared to control animals that were not infected with MCMV before. Thus, this study demonstrated systematically that vaccine- or infection-induced CD4+ T cell responses may be harnessed to modulate the immune response against an antigen of choice using T helper liposomes [183].

These findings were later translated into the HIV-1 vaccine context. Mice were immunized first with OT-II peptide and then boosted with Env/OT-II-liposomes. A similarly modulated phenotype of the Env-specific humoral immune response was observed compared to mock-primed control groups or animals that received T helper liposomes with an irrelevant peptide encapsulated. The anti-Env antibody response could further be modulated by immunizing mice with DNA encoding the HBV surface antigen (HBsAg) and boosting with Env-liposomes that encapsulated an HBsAg-derived peptide, which strongly overlapped in its amino acid sequence with a promiscuous HBV T cell epitope described in a study from 1992 [184]. Surprisingly, this immunization regimen only improved Th2 responses [185].

More studies addressing ISH effects with LbNP vaccine candidates were performed with VLPs. In an initial *proof-of-concept* study, mice were primed with DNA coding for the HIV capsid precursor protein p55 (Gag) and boosted with lentiviral VLPs bearing Env on the surface and the HIV capsid core inside [186]. Here, ISH effects were mediated by CD4+ T cell responses specific for Gag. In subsequent studies, the system was translated to harnessing pre-existing CD4+ T cells elicited by licensed vaccines or clinical vaccine candidates to modulate HIV-specific humoral immune responses. Mice were immunized with the licensed Tetanus vaccine Tetanol^®^pur containing Tetanus Toxoid (TT) and boosted with lentiviral VLPs that incorporated the immunodominant TT peptide p30 [187]. Likewise, Klessing et al. performed priming immunizations with an experimental Tuberculosis vaccine (H1/CAF01), which contains epitopes from the antigens Ag85B and ESAT-6. These mice then received VLPs displaying Env on the surface and incorporating a Gag-H1 fusion protein [188].

While Gag- and H1-mediated ISH strongly promoted Th1-type anti-Env IgG2a/c subtype responses with antiviral Fc effector functions, the Tetanus Toxoid-mediated system predominantly increased anti-Env IgG1 antibodies. Notably, the adjuvant system of the licensed vaccine plays a crucial role in the Th polarization that is imprinted on the Env-specific immune response. On an interesting side note, the study describing the Tuberculosis vaccine-mediated ISH also demonstrated a correlation between ISH effects and higher numbers of Env-specific long-lived plasma cells [188]. Additionally, the high-titer Env-specific IgG subtype levels shaped by ISH in this study demonstrated durable longevity with a plateau-like progression up to week 24 after the last nanoparticle boost in the murine model.

A mechanism related to ISH is the so-called intramolecular help (IMH). Here, the main immunogen is recombinantly fused with a helper peptide or protein. This does not require a nanoparticulate delivery and, thus, is beyond the scope of this review. Just to give two examples, Narayanan et al. fused the major timothy grass pollen allergen (Phl p 1) with an allergen-unrelated epitope to induce allergen-specific IgE antibody responses without cognate T cell help, which may path the way for novel allergy vaccines [189]. Ng et al.—like Klessing et al. mentioned above-harnessed an epitope derived from the *Mycobacterium tuberculosis* antigen Ag85B to modulate humoral immune responses via IMH. They primed mice with *M. bovis* bacille Calmette–Guérin (BCG), which also comprises the Ag85B protein. The animals were boosted with immunogens (OVA and Ebola virus glycoprotein (EBOV-GP)) fused to an immunodominant CD4+ T cell epitope derived from Ag85B. The induction of IMH resulted in an isotype switching to IgG2c in both anti-OVA and anti-EBOV-GP antibody responses. Additionally, promoted anti-EBOV-GP IgG1 demonstrated a high affinity to the antigen as well as neutralizing activity. The authors further observed the dose-sparing effects of BCG-specific Tfh cells on the phenotypic outcome of IMH, which might also be relevant for ISH [190].

Until now, no study has directly compared the modulation of immune responses following immunization with TLR agonist-adjuvanted LbNPs vs. T helper LbNPs in the context of ISH. However, in a recent study,, we performed immunizations of mice with inorganic calcium phosphate (CaP) nanoparticle-based vaccines. Animals previously immunized with Tetanol^®^pur received HIV-1 Env trimer-conjugated CaP nanoparticles either with encapsulated p30 peptide derived from TT (Env-CaP-p30) or with encapsulated TLR9 ligand CpG (Env-Cap-CpG). Both Env-CaP-p30 and Env-Cap-CpG induced comparable levels of total anti-Env IgG, although the anti-Env IgG subtype distribution varied between the groups [191]. These results clearly demonstrated that the ISH strategy for nanoparticle-based vaccines might indeed efficiently substitute excessive TLR stimulation.

Taken together, both ISH and IMH are nifty mechanisms that harness loopholes in the immune system to enhance and modulate antibody responses without the need for additional TLR agonists as adjuvant. Based on the vaccination status of the population in different parts of the world, ISH-based nanoparticulate vaccine candidates may be tailored to encapsulate matching CD4+ T cell epitopes. Especially in countries that struggle with a high HIV-1 incidence and regular EBOV outbreaks, pre-existing BCG-specific cellular immunity may be harnessed to generate effective vaccines against those viruses. However, more systematic research needs to be performed on the role of Tfh cells that provide ISH and the dose-dependency of encapsulated T cell epitopes and/or displayed antigens on in vivo effects.

### 3.2. Universal T Cell Epitopes

Recombinant nanoparticle vaccines based on a single protein may elicit only suboptimal T cell help, which results in a poor antibody response. This problem cannot be completely solved by the incorporation of TLR ligands only [192]. The introduction of a T helper cell epitope might increase MHC-II-restricted responsiveness and the magnitude and affinity of the antibody responses [192,193].

**Figure 4:**
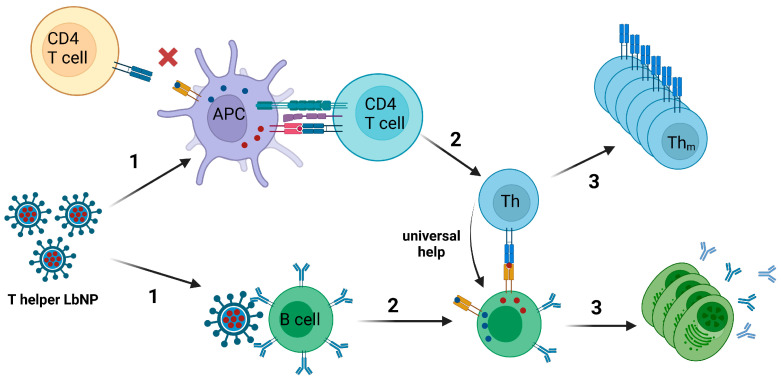
Universal T cell epitopes. T helper LbNPs carry viral glycoproteins, which elicit no or suboptimal T cell help on the surface and encapsulate universal T cell epitopes. (1) Upon priming with those T helper LbNPs, naïve CD4+ T cells recognize these universal epitopes complexed on MHC-II molecules of APCs, which leads to proliferation and differentiation of the CD4+ T helper cells. Simultaneously, glycoprotein-specific, naïve B cells take up LbNPs in a BCR-dependent manner. (2) After processing the LbNPs in the B cells, the encapsulated epitopes are presented on the MHC-II molecules of these B cells. These epitope/MHC-II complexes are subsequently recognized by the simultaneously induced universal T helper cells (Th). (3) After the immune reaction is resolved, elicited universal T helper memory cells (Th_m_) could be further recruited via the ISH mechanism (see Figure 3). Created with BioRender.com.

The co-evolution of humans and pathogens like *Clostridium tetani, Corynebacterium diphteriae*, or various herpesviruses resulted in the presence of naïve T cell clones within the total T cell repertoire that may be triggered by pathogen-derived peptide epitopes even though the host organism never had a vaccination against or contact with the respective pathogen [194]. These stimulating epitopes are called “universal T cell epitopes” [195]. Especially in the late 20th century, multiple studies screened such epitopes derived from Tetanus Toxoid or Diphtheria Toxoid [196,197,198]. Importantly, these identified universal T cell epitopes may be encapsulated into nanoparticulate vaccines to induce ISH-like effects without pre-existing cellular immunity against the peptides (Figure 4).

In 1991, Garcon et al. described a “thymus-dependent” liposomal vaccine to provide universal T cell help for weak antigens. They co-incorporated a lipophilic hapten (DNP-aminocaproyl phosphatidylethanolamine) and a helper peptide derived from hemagglutinin of influenza A virus (HA2) into liposomes. Mice that were immunized with hapten/HA2-liposomes showed an improved hapten-specific antibody response with a multitude of IgG subclasses elicited and also a memory response for the hapten. The control group that received hapten-liposomes without helper peptides only showed a hapten-specific IgM response. Furthermore, the authors demonstrated that the liposomes must be intracellularly processed in order to elicit the modulated immune response phenotype [199]. In the same line, Boeckler et al. designed liposomal di-epitope constructs that allowed the physical combination of B and “universal” T cell epitopes as structurally separate entities within the same vesicle. Liposomal preparations that carried B and T cell epitopes on the same vesicles were very effective in generating strong antibody responses against the B cell epitope, characterized by high titers and by a long duration. Moreover, the authors demonstrated the importance of the association of the two epitopes within a single vesicle [200].

In a more recent study, the encapsulation of a TT-derived peptide (p30) in nanoparticles displaying HIV-1 Env significantly enhanced the anti-Env antibody response in mice compared to animals that were immunized with Env-coupled nanoparticles only [191].

Similarly, Fraser et al. designed LbNP-like water-in-oil nanoparticles that were coupled with nicotine on the surface and encapsulated a chimeric MHC-II-restricted peptide containing epitopes from Tetanus Toxoid and Diphtheria Toxoid (TpD) [201]. These T helper nanoparticles strongly increased anti-nicotine humoral immune responses in non-human primates compared to control nanoparticles without TpD. Notably, all nanoparticle formulations in this study additionally contained the TLR7/TLR8-agonist R-848 (resiquimod).

Hanson et al. formulated liposomes displaying HIV MPER peptides on the surface and incorporating two universal T cell epitopes in the vesicle walls: LACK1 derived from *Leishmania infantum* and HIV30 derived from the HIV Env subunit gp120. The T cell help elicited by these liposomes resulted in an enhancement of anti-MPER antibody responses in a comparable range to MPLA- or CpG-supplemented MPER-liposomes [109]. This study and a subsequent study using an identical liposome formulation (MPER/LACK1-liposomes) further compared immunological effects of LACK1 anchored on compounds of the lipid bilayer and, thus, being displayed in the liposomal cavity, but also alongside MPER on the nanoparticle surface (pLACK), and of soluble LACK1 peptides in the aqueous cavity (sLACK). The data demonstrated that sLACK resulted in improved MPER-specific germinal center (GC) B cell formation, probably because the humoral immune system is less distracted when the antigen of interest alone is displayed on the liposomal surface and the T helper peptides are hidden inside. On the other hand, pLACK induced a higher frequency of LACK1-specific GC B cells. MPER-specific antibody titers were (not significantly) higher with sLACK, but pLACK induced anti-MPER antibodies with higher affinity [202].

Another example of universal T cell peptides is the pan(-HLA)-DR-binding epitope (PADRE) [203]. This 13-aa-long peptide binds with high affinity to the most common human HLA-DR types and, thus, overcomes HLA-DR polymorphisms [204]. One publication indicates that PADRE is up to 100-fold more potent in inducing human T cell proliferation than Tetanus-derived universal T cell epitopes [196]. In a clinical phase-1 study, PADRE was shown to be well-tolerated and safe [205]. Moreover, a PADRE-derivatized dendrimer complexed with amphotericin B (AmB) on liposomes as a therapeutic vaccine against cutaneous leishmaniasis enhanced drug efficacy by 83% and reduced AmB toxicity via dose-sparing effects [206]. Another liposomal vaccine combining PADRE with HER2/neu-derived peptides AE36 and E75 resulted in a reduction of tumor growth via induction of potent CD8+ T cell responses in a mouse model for breast cancer [207].

Taken together, the incorporation of universal T cell epitopes into nanoparticulate vaccine formulations is an elegant way to harness an evolution-shaped epitope selection to improve humoral immune responses against the immunogen of choice in an ISH/IMH-like fashion. On a critical side note and also relevant for ISH/IMH, most of the studies mentioned above were performed in inbred animal systems. Here, identified immunodominant T cell epitopes are hypothetically capable of restimulating CD4+ T cell populations with 100% coverage among the animals of a cohort. The situation in humans with a multitude of MHC-II alleles is different. One immunodominant peptide encapsulated in liposomes might not be enough to induce a broad coverage of pre-existing T cell restimulation in the population. This may require screenings of peptide libraries to identify a combination of T helper cell epitopes that may mediate ISH effects with sufficient coverage. Alternatively, whole heterologous antigens need to be encapsulated in the nanoparticles, which will then be processed into a multitude of polyclonal T helper epitopes.

Once these challenges are overcome, the harnessing of heterologous cellular immunity to modify and improve antibody responses against weak immunogens from threatening pathogens may alter the face of vaccinology in the current century and increase vaccine acceptance due to a lack of adjuvants in the formulation.

## 4. Conclusions and Future Perspectives

The development of vaccines has traditionally been an empirical process, relying on trial and error to identify effective formulations. While this approach has yielded numerous life-saving vaccines, it often lacks the precision and predictability needed to address emerging pathogens and optimize vaccine efficacy. The current landscape of vaccine development highlights a transition from empirically designed to rationally designed vaccines, guided by a deeper understanding of the immune system and the mechanisms underlying protective immune responses.

In this review, we contrasted two distinct approaches: the incorporation of TLR ligands as triggers of innate immunity or heterologous/universal T cell epitopes that harness a loophole in the adaptive immune system. Both approaches, however, ultimately lead to the same aim: increasing the immunogenicity of lipid-based vaccines.

Conventional adjuvants, such as alum salts, have been developed empirically and used for decades to enhance the immunogenicity of vaccines. However, their redundant mode of action remains incompletely elucidated, making it challenging to tailor their effects “on demand”. In contrast, TLR agonists offer greater control over immune activation by targeting defined pathways. This enables the fine-tuning of vaccine-induced responses to achieve improved protection and facilitates the optimization of vaccine formulations for enhanced efficacy and safety.

While TLR agonists primarily target innate immunity, intrastructural and intramolecular help, as well as the incorporation of universal T helper cell epitopes, represent novel strategies to engage the adaptive arm of the immune system. Due to a lack of immunodominant epitopes, recombinant protein vaccines may elicit suboptimal T cell help despite appropriate adjuvantation. Recruitment of pre-existing, heterologous T cell responses via ISH/IMH efficiently accelerates the kinetics and magnitude of antibody responses. Alternatively, the incorporation of universal T cell epitopes into nanoparticulate vaccine formulations can effectively substitute cognate T cell help.

Notably, the advancement of lipid-based vaccine formulations (including mRNA-LNPs) represents a promising avenue, offering a versatile platform for the incorporation of these diverse strategies. The potential future of lipid-based vaccine development may lie in the integration of these approaches to bridge the gap between innate and adaptive immunity. By combining TLR agonists and adaptive immunity-harnessing strategies, it might be possible to capitalize on the strengths of each approach, leading to a comprehensive and synergistic enhancement of vaccine efficacy.
